# Metabolic Profiling of Central Nervous System Disease in *Trypanosoma brucei rhodesiense* Infection

**DOI:** 10.1093/infdis/jix466

**Published:** 2017-09-12

**Authors:** Sabrina D Lamour, Vincent P Alibu, Elaine Holmes, Jeremy M Sternberg

**Affiliations:** 1 Department of Infectious Disease Epidemiology, School of Public Health; 2 Section of Biomolecular Medicine, Division of Computational and Systems Medicine, Department of Surgery and Cancer, Imperial College London, London; 3 Institute of Biological and Environmental Sciences, University of Aberdeen, Aberdeen, United Kingdom; 4 Department of Biochemistry, Makerere University, Kampala, Uganda

**Keywords:** Human African trypanosomiasis, CNS, neurological symptoms, metabolic profiling, nuclear magnetic resonance

## Abstract

**Background:**

The progression of human African trypanosomiasis from the early hemolymphatic stage to the late meningoencephalitic stage is of critical diagnostic importance as it determines the choice of potentially toxic drug regimens. Current diagnostic criteria involving analysis of cerebrospinal fluid (CSF) for parasites and/or pleocytosis are sensitive, but recent evidence suggests that specificity may be poor.

**Methods:**

We used an untargeted global metabolic profiling approach for the discovery of novel candidate stage-diagnostic markers in CSF from patients infected with *Trypanosoma brucei rhodesiense*, using ^1^H nuclear magnetic resonance (NMR) spectroscopy.

**Results:**

Metabolic markers did not distinguish between early and late-stage cases but were associated with neuroinflammatory responses and the presentation of neurological disturbances. In particular, increased concentrations of 3-hydroxybutyrate and alanine and reduced concentrations of mannose and urea were discriminatory for the presentation of daytime somnolence and gait ataxia.

**Conclusions:**

CSF metabolite concentrations provide markers for neuroinflammatory responses during central nervous system (CNS) invasion by trypanosomes and are associated with the presentation of neurological disturbances independently of disease stage determined by current criteria. This suggests that applying a dichotomous-stage diagnosis on the basis of CSF pleocytosis does not accurately reflect the biological changes occurring as parasites invade the CNS and has implications for biomarker discovery strategies.

## Teaser

^1^H nuclear magnetic resonance metabolic profiling of cerebrospinal fluid demonstrated that biochemical markers were associate with presentation of neurological symptoms in African trypanosomiasis, rather than disease stage. Thus, disease staging using standard diagnostic methods may not accurately mirror the invasion of the brain by parasites.

Human African trypanosomiasis (HAT) is caused by infection with the tsetse fly–transmitted hemoflagellates *Trypanosoma brucei rhodesiense* and *Trypanosoma brucei gambiense*. After an initial hemolymphatic infection, parasites penetrate the central nervous system (CNS), leading to meningoencephalitis, which is fatal if untreated [1]. Progression from the hemolymphatic to meningoencephalitic stage presents a therapeutic challenge because of the toxic nature of drugs required once the parasites are present in the CNS. In *T. b. rhodesiense* infection, treatment requires the use of the trivalent arsenical melarsoprol, with serious neurological damage ensuing in up to 10% of treatments and a fatal reactive encephalopathy in up to 5% of treatments. In *T. b. gambiense* infection, melarsoprol treatment of the late stage has been largely replaced with the better-tolerated eflornithine and nifurtimox combination therapy, but this treatment still carries significant side effects in up to 2% of treatments and is logistically challenging to deliver [2].

Because of the hazards involved with treatment of the late stage of infection, an accurate diagnosis of stage is essential. Currently this involves detection of one or both of the parasites in the cerebrospinal fluid (CSF) or a pleocytosis of the CSF >5 cell/mm^3^ [3]. However, these diagnostic criteria are now under question. In *T. b. gambiense* infection, it has been demonstrated that moderate pleocytosis of the CSF, from 5 cells/µL to 20 cells/µL, may still be effectively treated with drugs developed for early stage infection [4]. In *T. b. rhodesiense* HAT, neuroinflammatory responses symptomatic of parasite invasion of the brain have been detected in cases that were also effectively treated with early stage drugs [5]. Furthermore, neurological symptoms, such as somnolence, which formerly were considered to be exclusive evidence of the late stage of infection, have been detected in a proportion of patients with early stage infection [6]. These clinical observations are consistent with those from studies of CNS invasion in experimental rodent models of HAT in which initial invasion takes place very early in infection and at a time when animals may still be effectively treated with early stage drugs [7, 8]. Taken together, the clinical and experimental observations indicate that the current staging criteria do not accurately reflect disease progression in the brain and that new, biologically and therapeutically relevant staging criteria are required in HAT.

Metabolic profiling has been shown to be a powerful tool in providing key insights into the complex dynamic interplay between metabolic processes of both the host and the parasite, where parasite-related perturbations in host metabolic pathways may highlight different mechanisms involved in either acute or chronic infection [9]. We have previously used this approach to investigate plasma from clinical *T. b. rhodesiense* HAT cases and in experimental animal models to discover disease diagnostic markers and understand pathophysiological processes [10, 11]. A similar approach has been described using untargeted liquid chromatography mass spectrometry metabolic screening of plasma, urine, and CSF specimens from patients infected with *T. b. gambiense* [12]. We now describe the analysis of CSF from patients with *T. b. rhodesiense* HAT, using proton nuclear magnetic resonance (^1^H NMR) spectroscopy, a high-throughput and robust method that is optimal for analyzing multiple metabolites simultaneously within complex biological mixtures [13, 14], including CSF [15]. Cytokine concentrations in CSF samples were additionally measured as markers of local inflammation and were correlated with metabolite levels.

The aim of this study was to determine the relationship of small-metabolite markers to current staging criteria, neuroinflammatory processes, and the presentation of neurological symptoms as a strategy to discover novel diagnostic markers and understand the physiological processes involved in progression of HAT.

## METHODS

### Ethics Statement

Human CSF samples were collected from adult patients with suspected HAT only, following standard diagnostic methods [16] and approved by ethics committees in Uganda (Uganda National Council for Science and Technology reference no. 082786) and the United Kingdom (North of Scotland Research Ethics Committee reference no. 08/S0802/126), conforming to the principles of the Declaration of Helsinki. Ethical consent forms were designed in English and translated into local languages. Consent was given as a signature or a thumbprint after verbal explanation. For those aged <18 years, consent was given by the legal guardian.

### Patient Information and Sample Collection

Details of study sites, patient recruitment, and diagnostic and treatment protocols have been presented elsewhere [6, 11]. A total of 51 patients with HAT were recruited at Lwala Hospital, Kaberamaido District, and Serere Health Center, Serere District, in eastern Uganda, between November 2008 and March 2010. CSF samples were collected from all patients as part of normal diagnostic and staging procedures. Late-stage cases were identified using World Health Organization staging criteria, consisting of the presence of parasites in the lumbar CSF and/or a CSF white blood cell count of >5 cells/µL [16]. Aliquots of 1–2 mL of CSF were immediately frozen and then maintained in liquid nitrogen until transferred to the United Kingdom via airfreight (on dry ice for 24 hours) and stored at –80°C until analysis. Of the original 51 CSF samples, 46 were available to use for subsequent metabolic analyses. A summary of the parasitological, demographic, and neurological characteristics of the patients can be found in the results.

### Metabolic Data Acquisition and Processing via ^1^H NMR Spectroscopy

A total of 46 CSF samples from patients with HAT underwent metabolic screening via ^1^H NMR spectroscopy. Samples were prepared in 1 batch in a randomized order. Prior to acquisition, an aliquot of 280 µL per CSF sample was diluted with 260 µL of distilled water and 60 μL of Bruker urine buffer (1.5 M K_2_HPO_4_, 2 mM NaN_3_, 0.1% volume/volume 3-[trimethylsilyl]propionic-2,2,3,3-d4 acid sodium salt [TSP] solution, and 10% volume/volume D_2_O, with pH adjusted to 7.4; Sigma-Aldrich, Germany) in microfuge tubes. Samples were centrifuged for 1 minute at 14 000×*g*, and 570 µL per sample was transferred into 5-mm NMR tubes. ^1^H NMR data were acquired (over 3 days) on a Bruker Avance 600 MHz (14.1 T) NMR spectrometer with a BB probe head and refrigerated SampleJet autosampler (Bruker, Germany), using Topspin software (version 3.1, Bruker BioSpin, Germany). Metabolic data were acquired as described elsewhere [11] with the following modifications: acquisition via a standard 1-dimensional pulse and water suppression program was performed at 310 K, with 256 acquired free induction decays and 4 dummy scans. Data processing (including automatic phasing, baseline correction, TSP reference peak calibration, automatic spectral alignment, and probabilistic quotient normalization) were performed using MATLAB (version R2013a; Mathworks) and Topspin as outlined by Lamour et al [11]. Spectral assignments of metabolite peaks were identified using Chenomx NMR suite profiler 8.1 software and literature references [15, 17]. Spectral peaks derived from the drug metabolite paracetamol (for acetaminophen; 2.15 singlet [s] and 6.92 and 7.25 doublets [d]) and contaminant ethanol resonances (1.19 triplet [t] and 3.68 quadruplet [q]) were identified in a subset of samples, and these spectral regions were thus excluded from the entire data set.

### CSF Cytokine Analysis

Interferon γ (IFN-γ), interleukin 6 (IL-6), and interleukin 10 (IL-10) concentrations were measured using a solid-phase sandwich enzyme-linked immunosorbent assay (OptiEIA; BD Pharmingen), as described previously [18]. Biological limits of detection were 1.8, 8.3, and 1.6 pg/mL, respectively. When the cytokine concentration was lower than the lower limit of detection, a nominal concentration of 0.5 times the limit of detection was recorded.

### Data Analysis

Following data processing, spectral NMR files were analyzed using principal component analysis (PCA) and orthogonal partial least squares discriminatory analysis (OPLS-DA) (SIMCA software, version 14.0; Umetrics, Sweden). PCA is an unsupervised pattern-recognition model used to evaluate overall variance in the data set, while OPLS-DA is a supervised modeling method that maximizes differences between groups while minimizing within-group variation and was used to compare profiles for patients with early infection to those for patients with late-stage infection [19]. In both PCA and OPLS-DA, samples are schematically represented as coordinates on component planes and plotted on scores plots. Their relative positions to each other are dependent on their spectral similarity: samples that share similar patterns in their metabolic data (eg, those that have similar spectral peaks) are grouped together on the plot, whereas samples that show marked differences are segregated. The variables responsible for their positioning in the scores plot are shown in the corresponding loadings plot. This modeling strategy was repeated, using the integrals of endogenous metabolites identified via NMR, to discriminate differentiating metabolites between patients with neurological manifestations (ie, somnolence, abnormal gait, and tremor) and those without such manifestations or with a Glasgow coma scale score of <15 and to assess the impact of other potential confounding factors (ie, patient age and sex and sample collection site).

Metabolite integrals were further analyzed via univariate nonparametric tests, using Mann-Whitney *U* tests, followed by Benjamini-Hochberg false-discovery rate adjustment [20] to correct for multiple testing. Percentage differences between patients stratified by stage or presence of a neurological symptom were reported on the basis of the median integral values for each group relative to those for patients with early stage infection or without symptoms. Spearman-based correlation analysis was performed between cytokine concentrations and integrals of metabolites that were discriminatory for neurological disturbances, with Benjamini-Hochberg false-discovery rate adjustment.

## RESULTS

### Patients and Symptoms

Demographic and clinical details of patients with HAT recruited to this study are presented in Table 1. While neurological symptoms were observed mainly in patients with late-stage infection, patients with early stage infection also exhibited tremor and gait abnormalities.

**Table 1.** Demographic Characteristics and Central Nervous System (CSF) Symptoms Among Patients With Early or Late-Stage Human African Trypanosomiasis

Characteristic	Early Stage (n = 11)	Late Stage (n = 31)
Sex, male:female	3:8	14:17
Age, y, median (range)	25 (15–70)	26 (3–70)
CSF WBC count, cells/µL, median (IQR)	4 (3–4)	38 (15–119)
Coma^a^	0	6
Gait abnormality	1	16
Tremor	2	12
Somnolence	0	14

Abbreviations: IQR, interquartile range; WBC, white blood cell.

^a^Defined as a Glasgow coma scale score of <15.

Inflammatory and regulatory cytokine concentrations were also measured in a subset of CSF samples. These were selected purely on the basis of having sufficient residual volume to allow quadruplicate enzyme-linked immunosorbent assays and hence may be regarded as a random sample (comprising 8 of 11 early stage cases and 19 of 31 late-stage cases). Progression from early to late-stage infection was associated with significant increases in IL-6, IFN-γ, and IL-10 concentrations (Table 2).

**Table 2.** Cytokine Concentrations in Cerebrospinal Fluid (CSF) Samples From Patients With Early or Late-Stage Human African Trypanosomiasis

Stage	Concentration, pg/mL, Median (IQR)		
	IL-6	IFN-γ	IL-10
Early (n = 8^a^)	4.1 (4.1–4.1)^b^	0.9 (.9–11.5)^b^	15.7 (.8–87.0)
Late (n = 19^a^)	30.1 (2.8–185.4)^c^	25.1 (7.0–38.4)^d^	224.2 (87.3–515.3)^d^

Abbreviations: IFN-γ, interferon γ; IL-6, interleukin 6; IL-10, interleukin 10; IQR, interquartile range.

^a^Data are a subset of total samples because of limited CSF volumes.

^b^Data are reported as the lower limit of detection times 0.5.

^c^ *P* < .05 by the Mann-Whitney *U* test, compared with early stage cases

^d^ *P* < .01 by the Mann-Whitney *U* test, compared with early stage cases.

### Metabolic Phenotype in Early and Late-Stage HAT

The metabolic profiles in CSF samples collected from patients with HAT were screened using a standard 1-dimensional ^1^H NMR pulse program, a common method for detecting and quantifying a wide range of complex biological molecules. Using this technology, we identified the peaks of 27 endogenous metabolites, comprised largely of sugars, amino acids, amines, and organic acids, with relatively low levels of lipid and protein resonances (Supplementary Table 1). Two exogenous metabolites were also detected (ie, paracetamol/acetaminophen and ethanol peaks), which were removed from all spectra prior to analyses.

**Figure 1.**
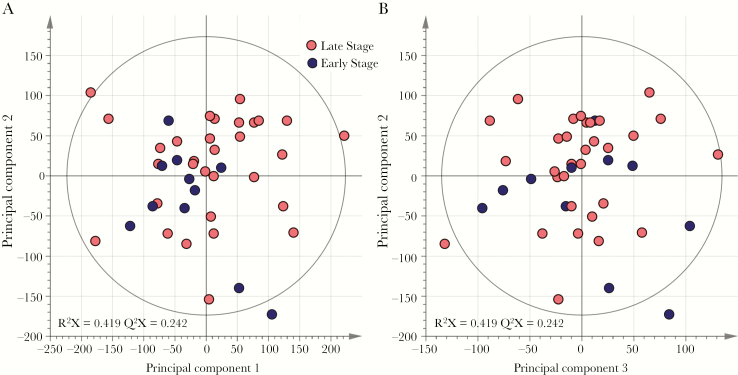
Inherent variation in cerebrospinal fluid (CSF) metabolic phenotypes is not associated with human African trypanosomiasis diagnostic stage. Principle component analysis scores plotted on the basis of ^1^H nuclear magnetic resonance spectral data of CSF samples show overlap of spectral data from patients with early stage disease (n = 11) and those with late-stage disease (n = 31), using the first 2 components (*A*) and the second and third components (*B*). R^2^X denotes the model fit parameter for variation in spectral data and describes the fraction of variance explained by model. Q^2^X denotes the model predictive parameter for spectral data and describes the predictive power of the model.

To discover metabolite patterns that may be associated with disease state, multivariate analysis of the NMR peak integral data was undertaken using PCA, followed by OPLS-DA. Results from multivariate analyses of NMR data demonstrated that the inherent variability within the metabolic phenotype of CSF samples was not associated with diagnostic stage classification of HAT. PCA models using either complete spectra (Figure 1) or integrals from the aforementioned 27 metabolites (Supplementary Figure 1) did not show any separation between the metabolic profiles from patients with early stage and those with late-stage infections, indicating that both groups display strong similarities in their metabolic profiles and that the variance within the sample set is not driven by stage of infection. Further comparisons between stages using discriminant multivariate analyses (ie, OPLS-DA [Supplementary Figure 1]), to enhance potential subtle differences between these groups, and univariate nonparametric tests (Supplementary Table 1) did not reveal any significant differences. Furthermore, no significant differences in metabolite signatures were observed with respect to patient sex, patient age, or sample collection site. PCA analyses revealed that inherent variance was largely attributed to differences in CSF concentrations of glutamine, creatinine, *myo*-inositol, and 3-hydroxyisovalerate.

### Metabolic Phenotype Discriminates Patients Exhibiting Neurological Symptoms

**Figure 2.**
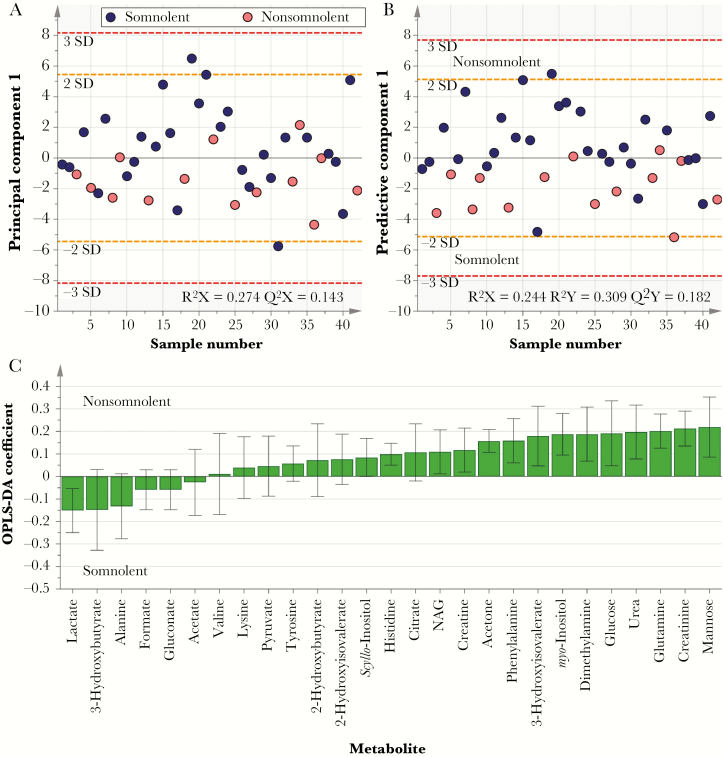
Metabolic changes in cerebrospinal fluid (CSF) associated with somnolence. *A*, Principle component analysis score plot, comparing metabolic phenotypes for somnolent patients (n = 14) to those for nonsomnolent patients (n = 28). *B* and *C*, Cross-validated orthogonal partial least squares discriminatory analyses (OPLS-DA) score plot (*B*) and corresponding loadings plot (*C*). Partial separation between the 2 groups along the first component is apparent across both models. NAG, *N*-acetyl glycoprotein.

PCA and OPLS-DA were used to investigate association between the CSF metabolic markers and the presence or absence of neurological symptoms in patients, including somnolence, gait impairment, tremor, and impairment of consciousness as measured by the Glasgow coma scale score. Differences in metabolic phenotypes were observed between somnolent and nonsomnolent patients, demonstrated by partial vertical separation between 2 groups along the first component in both models, although particularly in OPLS-DA (Figure 2). Examination of the corresponding OPLS-DA loadings plot revealed that levels of lactate and the ketone body 3-hydroxybutyrate (3-HB) were higher in patients displaying somnolence, who also had lower levels of a range of sugars, including mannose and glucose, and amines, such as glucosamine and dimethylamine, creatinine, and urea. Similar results were also observed for the presence or absence of gait abnormalities in patients, including higher levels of lactate and 3-HB and lower levels of mannose in patients with gait abnormalities, compared with those without gait impairment (Supplementary Figure 2). PCA did not reveal any distinction in metabolic profiles between patients with and those without tremors or an abnormal Glasgow coma scale score, nor were their corresponding discriminant OPLS-DA models predictive.

### Somnolence and Gait Abnormalities Marked by Alterations in Ketone Bodies and Reduced Levels of Mannose

Nonparametric analyses of individual metabolite concentrations confirmed results observed in multivariate analyses, with significant differences observed between patients presenting with and those presenting without somnolence and gait impairment, as shown in Table 3. Patients with abnormal gait displayed lower concentrations of *myo*-inositol than those with no gait impairment, whereas somnolent patients had decreased levels of acetone in their CSF than alert patients. Additionally, increases in 3-HB and alanine concentrations and decreases in mannose and urea concentrations were significant for both somnolent patients and those with gait abnormalities. No significant differences in metabolite concentrations were found when the presence of tremor or an abnormal Glasgow coma scale score were the grouping variables.

**Table 3.** Alterations in Metabolite Concentrations in Cerebrospinal Fluid (CSF) Samples From Patients With Neurological Symptoms

Metabolite	GCS Score <15		Gait		Tremors		Somnolence	
	Difference^a^	*P* ^b^	Difference^a^	*P* ^b^	Difference^a^	*P* ^b^	Difference^a^	*P* ^b^
2-HB	–2.3	NS	–6.7	NS	–11.0	NS	–12.2	NS
2-HIV	0.3	NS	–1.7	NS	–10.3	NS	–7.5	NS
3-HB	8.8	NS	17.2	.028	6.8	NS	11.6	.0351
3-HIV	–10.5	NS	–15.5	NS	–12.2	NS	–23.0	NS
Acetate	–24.2	NS	–17.5	NS	–10.7	NS	29.9	NS
Acetone	–12.4	NS	–7.1	NS	13.4	NS	–24.9	.034
Alanine	29.3	NS	24.2	.007	2.0	NS	24.0	.012
Citrate	6.8	NS	–10.5	NS	–5.6	NS	–7.4	NS
Creatine	–3.9	NS	–3.8	NS	–10.1	NS	–3.3	NS
Creatinine	–32.8	NS	–13.2	NS	–0.8	NS	–18.9	NS
Dimethylamine	–23.4	NS	–21.9	NS	0.4	NS	–28.6	NS
Formate	3.5	NS	2.3	NS	–2.8	NS	4.3	NS
Gluconate	–3.5	NS	2.3	NS	–2.8	NS	4.3	NS
Glucose	–50.7	NS	–27.7	NS	–8.2	NS	–28.5	NS
Glutamine	–34.8	NS	–19.9	NS	–13.2	NS	–21.0	NS
Histidine	–4.4	NS	–8.2	NS	–7.9	NS	–3.7	NS
Lactate	32.5	NS	36.0	.045	18.8	NS	22.0	NS
Lysine	0.6	NS	5.4	NS	0.7	NS	–2.0	NS
Mannose	–45.7	NS	–43.0	.003	–23.9	NS	–38.9	.008
*scyllo*-Inositol	–14.6	NS	–6.6	NS	3.4	NS	–20.1	NS
*myo*-Inositol	–42.7	NS	–26.4	.045	–19.9	NS	–16.7	NS
NAG	–20.9	NS	–5.0	NS	–2.5	NS	–6.5	NS
Phenylalanine	–8.2	NS	–14.5	NS	–14.3	NS	–10.5	NS
Pyruvate	20.0	NS	34.5	NS	14.6	NS	–15.6	NS
Tyrosine	4.3	NS	–3.0	NS	–7.1	NS	–1.2	NS
Urea	–34.5	NS	–39.7	.045	–22.3	NS	–44.7	.018
Valine	23.5	NS	6.9	NS	–2.2	NS	3.1	NS

Concentrations were determined via ^1^H nuclear magnetic resonance spectroscopy.

Abbreviations: GCS, Glasgow coma scale; HB, hydroxybutyrate; HIV, hydroxyisovalerate; NAG, *N*-acetyl glycoprotein; NS, not significant.

^a^Data are percentage differences relative to values for symptomless patients.

^b^By the Mann-Whitney *U* test with false-discovery rate correction.

### Metabolites Discriminatory for Neurological Symptoms Are Associated With CNS Immune Activation

**Figure 3.**
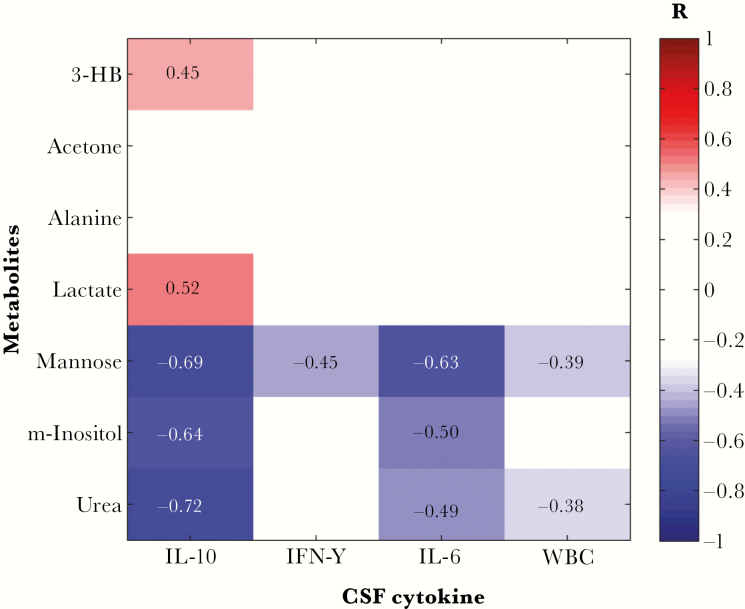
Metabolites discriminatory for patient neurological symptoms correlate with immunological measures in the central nervous system. False discovery rate–corrected Spearman-ranked correlation coefficient matrix shows statistically significant (*P* < .05) positive and negative correlations. 3-HB, 3-hydroxybutyrate; IL-6, interleukin 6; IL-10, interleukin 10; IFN-γ, interferon-γ; m-inositol, *myo*-inositol; WBC, white blood cell count.

CSF concentrations of IL-10, IL-6, and IFN-γ were significantly increased in patients with late-stage infection, compared with those with early stage infection (Table 2). We therefore examined whether there was an association between any of the metabolic markers discriminatory for neurological disturbances and immune activation. Several metabolites that had been shown to have significantly altered levels in patients presenting with somnolence and/or gait abnormalities exhibited significant relationships with markers of CNS immune activation (Figure 3). Both IL-10 and IL-6 were negatively correlated with mannose, *myo*-inositol, and urea, with IL-10 being additionally positively correlated with 3-HB and lactate. Interestingly, mannose was found to be significantly negatively correlated with all 3 cytokines, as well as with white blood cell count.

## DISCUSSION

In this study, an untargeted ^1^H NMR metabolic profiling approach was used to characterize the small-metabolite profiles in CSF samples for patients with HAT in different stages of disease progression. It was anticipated that these would inform discovery programs for new diagnostic biomarkers and provide candidate metabolic pathways associated with the neuropathogenesis of this disease. However, no metabolites were detected that could distinguish early from late-stage cases. Instead, a subset of metabolites was found to be discriminatory for patients exhibiting neurological signs of infection. In particular, patients presenting with both daytime somnolence and gait abnormalities exhibited higher levels of 3-HB and alanine and reduced levels of mannose and urea. Additionally, patients presenting with either of these symptoms also exhibited higher CSF concentrations of lactate and lower levels of *myo*-inositol and acetone. These differences appear to be independent to those of plasma metabolite profiles previously described [11], where alanine concentrations, for instance, were lower in patients than controls, and *myo*-inositol levels were higher.

The mechanisms underpinning these metabolite changes are not defined and are likely to be complex and related to the activation of the cellular immune response in the CNS. In this respect, the relationships demonstrated between metabolites discriminatory for neurological symptoms and CSF inflammatory cytokines provide further evidence for the role of immunological disturbance in HAT neuropathogenesis. Progression in HAT has previously been shown to be associated with increases in levels of inflammatory cytokines, as well as the counterinflammatory cytokine IL-10 [5], as was confirmed in this study. The close correlation of IL-6 and IL-10 responses has also been observed in rodent infection models [21] and has been proposed to reflect the operation of an immunological homeostatic feedback mechanism in the brain [22]. Mannose, *myo*-inositol, and urea, all negatively associated with neurological disturbance, were found to be also significantly negatively correlated with CNS IL-6 and IL-10 responses. Similarly, 3-HB and lactate, which were positively associated with neurological disturbance, were also positively correlated with the CSF IL-10 concentration. This represents the first evidence linking neurological symptoms, the immune response, and metabolite changes in the CNS during infection.

Changes observed with ketone bodies such as 3-HB and acetone, alanine, and/or *myo*-inositol concentrations in CSF have been shown to be discriminatory for other neuroinflammatory diseases, including meningitis [23, 24] and hepatic encephalopathy [25, 26], but are also seen in other neurodegenerative diseases, such as Parkinson disease [27]. The large reduction in urea concentration associated with neurological symptoms might reflect changes in macrophage arginine metabolism resulting from a parasite-driven type 1 activation [28, 29].

The lack of clear discrimination of disease stage by CSF metabolites is consistent with our recent observations of kynurenine pathway activation in the CNS [30] and with a recent untargeted liquid chromatography mass spectrometry study of CSF from patients infected with *T. b. gambiense*, where metabolic signatures were only discriminatory between early and an advanced late stage of disease defined by CSF white blood cell concentrations of >20 cells/µL [12]. Thus, metabolic alterations and neuroinflammatory activation appear to be predictive of neurological sequelae in HAT but not of diagnostic stage. This highlights a major challenge for biomarker discovery in HAT staging and raises the question of whether the current empirically derived CSF white blood cell count diagnostic criteria can effectively be used as an objective reference standard in such work.

While a range of CSF cytokines [31], chemokines [32], and metabolites [33] have demonstrated stage discriminatory potential, a common problem is a lack of specificity. This may be due to the onset of CNS inflammatory activity shortly after parasite infection (ie, during early stage infection as defined by current criteria). This idea is supported by our observations of CNS immune activation in *T. b. rhodesiense*–infected patients who were correctly identified as having early stage infection, based on current diagnostic criteria (as they were treated successfully with suramin, without relapse), and yet who presented neurological sequelae, including impairment of consciousness [5]. Thus, functional disturbances in the CNS as evidenced here by small-metabolite perturbations and neurological symptoms may precede the onset of late-stage infection as defined by current diagnostic criteria, and conversely it is possible that those cases defined as late-stage infections but not exhibiting CNS metabolite changes and neurological symptoms might be amenable to early stage drug treatment. Such fluidity in stage progression would be consistent with recent studies in animal models in which trypanosomes locate in the CNS very early after experimental infection [7, 34].

In conclusion, we have used metabolic profiling to reveal subtle changes in CSF during disease progression in HAT. CSF metabolites correlated with neuroinflammation and the presentation of neurological disturbances independently of disease stage. This suggests that the currently used dichotomous-stage diagnosis based on CSF pleocytosis does not accurately reflect the biological changes occurring as parasites invade the CNS and indicates the potential of metabolic profiling in new biomarker discovery strategies.

## Supplementary Data

Supplementary materials are available at *The Journal of Infectious Diseases* online. Consisting of data provided by the authors to benefit the reader, the posted materials are not copyedited and are the sole responsibility of the authors, so questions or comments should be addressed to the corresponding author.

## Supplementary Material

Supplementary Figure_S1

Supplementary Figure_S2

Supplementary Table

## Notes

***Acknowledgments.*** We thank Isabel Garcia-Perez and Maria Lopez-Gonzales, for performing additional mass spectrometry analyses at Imperial College London.

***Financial support.*** This work was supported by the Medical Research Council MRC; (to S. D. L.), and Imperial College (MRC doctoral training award G1000390 to S. D. L.), and the Wellcome Trust (grant 082786 to J. M. S. and V. P. A.).

***Potential conflicts of interest.*** All authors: No reported conflicts. All authors have submitted the ICMJE Form for Disclosure of Potential Conflicts of Interest. Conflicts that the editors consider relevant to the content of the manuscript have been disclosed.

## References

[1] Sternberg JM, Maclean L A spectrum of disease in human African trypanosomiasis: the host and parasite genetics of virulence. Parasitology 2010; 137:2007–15. 20663245 10.1017/S0031182010000946

[2] Alirol E, Schrumpf D, Amici Heradi J Nifurtimox-eflornithine combination therapy for second-stage gambiense human African trypanosomiasis: Médecins Sans Frontières experience in the Democratic Republic of the Congo. Clin Infect Dis 2013; 56:195–203. 23074318 10.1093/cid/cis886

[3] Kennedy PGE Diagnosing central nervous system trypanosomiasis: two stage or not to stage? T Roy Soc Trop Med H 2008; 102:306–7. 10.1016/j.trstmh.2007.11.011 18187172

[4] Chappuis F, Loutan L, Simarro P, Lejon V, Büscher P Options for field diagnosis of human african trypanosomiasis. Clin Microbiol Rev 2005; 18:133–46. 15653823 10.1128/CMR.18.1.133-146.2005 PMC544181

[5] MacLean L, Reiber H, Kennedy PG, Sternberg JM Stage progression and neurological symptoms in *Trypanosoma brucei rhodesiense* sleeping sickness: role of the CNS inflammatory response. PLoS Negl Trop Dis 2012; 6:e1857. 23145191 10.1371/journal.pntd.0001857 PMC3493381

[6] MacLean LM, Odiit M, Chisi JE, Kennedy PG, Sternberg JM Focus-specific clinical profiles in human African Trypanosomiasis caused by *Trypanosoma brucei rhodesiense*. PLoS Negl Trop Dis 2010; 4:e906. 21151878 10.1371/journal.pntd.0000906 PMC2998431

[7] Frevert U, Movila A, Nikolskaia OV Early invasion of brain parenchyma by African trypanosomes. PLoS One 2012; 7:e43913. 22952808 10.1371/journal.pone.0043913 PMC3432051

[8] Myburgh E, Coles JA, Ritchie R In vivo imaging of trypanosome-brain interactions and development of a rapid screening test for drugs against CNS stage trypanosomiasis. PLoS Negl Trop Dis 2013; 7:e2384. 23991236 10.1371/journal.pntd.0002384 PMC3749981

[9] Holmes E, Wilson ID, Nicholson JK Metabolic phenotyping in health and disease. Cell 2008; 134:714–7. 18775301 10.1016/j.cell.2008.08.026

[10] Wang Y, Utzinger J, Saric J Global metabolic responses of mice to *Trypanosoma brucei brucei* infection. Proc Natl Acad Sci U S A 2008; 105:6127–32. 18413599 10.1073/pnas.0801777105 PMC2329718

[11] Lamour SD, Gomez-Romero M, Vorkas PA Discovery of Infection Associated Metabolic Markers in Human African Trypanosomiasis. PLoS Negl Trop Dis 2015; 9:e0004200. 26505639 10.1371/journal.pntd.0004200 PMC4624234

[12] Vincent IM, Daly R, Courtioux B Metabolomics Identifies Multiple Candidate Biomarkers to Diagnose and Stage Human African Trypanosomiasis. PLoS Negl Trop Dis 2016; 10:e0005140. 27941966 10.1371/journal.pntd.0005140 PMC5152828

[13] Keun HC, Ebbels TM, Antti H Analytical reproducibility in (1)H NMR-based metabonomic urinalysis. Chem Res Toxicol 2002; 15:1380–6. 12437328 10.1021/tx0255774

[14] Dumas ME, Maibaum EC, Teague C Assessment of analytical reproducibility of 1H NMR spectroscopy based metabonomics for large-scale epidemiological research: the INTERMAP Study. Anal Chem 2006; 78:2199–208. 16579598 10.1021/ac0517085 PMC6561113

[15] Wishart DS, Lewis MJ, Morrissey JA The human cerebrospinal fluid metabolome. J Chromatogr B 2008; 871:164–73. 10.1016/j.jchromb.2008.05.001 18502700

[16] World Health Organization. Control and surveillance of African trypanosomiasis. WHO Tech Rep Ser 1998; 881:1–113. 10070249

[17] Sweatman BC, Farrant RD, Holmes E, Ghauri FY, Nicholson JK, Lindon JC 600 MHz 1H-NMR spectroscopy of human cerebrospinal fluid: effects of sample manipulation and assignment of resonances. J Pharm Biomed Anal 1993; 11:651–64. 8257730 10.1016/0731-7085(93)80171-v

[18] Maclean L, Odiit M, Macleod A Spatially and genetically distinct African Trypanosome virulence variants defined by host interferon-gamma response. J Infect Dis 2007; 196:1620–8. 18008245 10.1086/522011 PMC2082664

[19] Eriksson L, Antti H, Gottfries J Using chemometrics for navigating in the large data sets of genomics, proteomics, and metabonomics (gpm). Anal Bioanal Chem 2004; 380:419–29. 15448969 10.1007/s00216-004-2783-y

[20] Benjamini Y, Hochberg Y Controlling the False Discovery Rate - a Practical and Powerful Approach to Multiple Testing. J Roy Stat Soc B Met 1995; 57:289–300.

[21] Sternberg JM, Rodgers J, Bradley B, Maclean L, Murray M, Kennedy PG Meningoencephalitic African trypanosomiasis: Brain IL-10 and IL-6 are associated with protection from neuro-inflammatory pathology. J Neuroimmunol 2005; 167:81–9. 16054238 10.1016/j.jneuroim.2005.06.017

[22] Sternberg JM Human African trypanosomiasis: clinical presentation and immune response. Parasite Immunol 2004; 26:469–76. 15771682 10.1111/j.0141-9838.2004.00731.x

[23] Coen M, O’Sullivan M, Bubb WA, Kuchel PW, Sorrell T Proton nuclear magnetic resonance-based metabonomics for rapid diagnosis of meningitis and ventriculitis. Clin Infect Dis 2005; 41:1582–90. 16267730 10.1086/497836

[24] Subramanian A, Gupta A, Saxena S Proton MR CSF analysis and a new software as predictors for the differentiation of meningitis in children. NMR Biomed 2005; 18:213–25. 15627241 10.1002/nbm.944

[25] Kreis R, Ross BD, Farrow NA, Ackerman Z Metabolic disorders of the brain in chronic hepatic encephalopathy detected with H-1 MR spectroscopy. Radiology 1992; 182:19–27. 1345760 10.1148/radiology.182.1.1345760

[26] Weiss N, Barbier Saint Hilaire P, Colsch B Cerebrospinal fluid metabolomics highlights dysregulation of energy metabolism in overt hepatic encephalopathy. J Hepatol 2016; 65:1120–30. 27520878 10.1016/j.jhep.2016.07.046

[27] Öhman A, Forsgren L NMR metabonomics of cerebrospinal fluid distinguishes between Parkinson’s disease and controls. Neurosci Lett 2015; 594:36–9. 25817365 10.1016/j.neulet.2015.03.051

[28] Rath M, Müller I, Kropf P, Closs EI, Munder M Metabolism via Arginase or Nitric Oxide Synthase: Two Competing Arginine Pathways in Macrophages. Front Immunol 2014; 5:532. 25386178 10.3389/fimmu.2014.00532 PMC4209874

[29] Namangala B, De Baetselier P, Noël W, Brys L, Beschin A Alternative versus classical macrophage activation during experimental African trypanosomosis. J Leukoc Biol 2001; 69:387–96. 11261785

[30] Sternberg JM, Forrest CM, Dalton RN Kynurenine pathway activation in human African trypanosomiasis. J Infect Dis 2017; 215:806–12. 28013248 10.1093/infdis/jiw623 PMC5388295

[31] Maclean L, Odiit M, Sternberg JM Intrathecal cytokine responses in Trypanosoma brucei rhodesiense sleeping sickness patients. Trans R Soc Trop Med Hyg 2006; 100:270–5. 16343570 10.1016/j.trstmh.2005.03.013

[32] Hainard A, Tiberti N, Robin X A combined CXCL10, CXCL8 and H-FABP panel for the staging of human African trypanosomiasis patients. PLoS Negl Trop Dis 2009; 3:e459 19554086 10.1371/journal.pntd.0000459 PMC2696178

[33] Tiberti N, Lejon V, Hainard A Neopterin is a cerebrospinal fluid marker for treatment outcome evaluation in patients affected by *Trypanosoma brucei gambiense* sleeping sickness. PLoS Negl Trop Dis 2013; 7:e2088. 23469311 10.1371/journal.pntd.0002088 PMC3585011

[34] Laperchia C, Palomba M, Seke Etet PF *Trypanosoma brucei* Invasion and T-Cell Infiltration of the Brain Parenchyma in Experimental Sleeping Sickness: Timing and Correlation with Functional Changes. PLoS Negl Trop Dis 2016; 10:e0005242. 28002454 10.1371/journal.pntd.0005242 PMC5217973

