# Supplementary material for: Metabolic Profiling of Central Nervous System Disease in Trypanosoma brucei rhodesiense Infection
Source: J Infect Dis. 2017 Sep 12;216(10):1273–80. doi: 10.1093/infdis/jix466 (PMC5853393; doi:10.1093/infdis/jix466)
Supplement: Supplementary Figure_S1 [file jix466_suppl_figure_s1.docx]

**
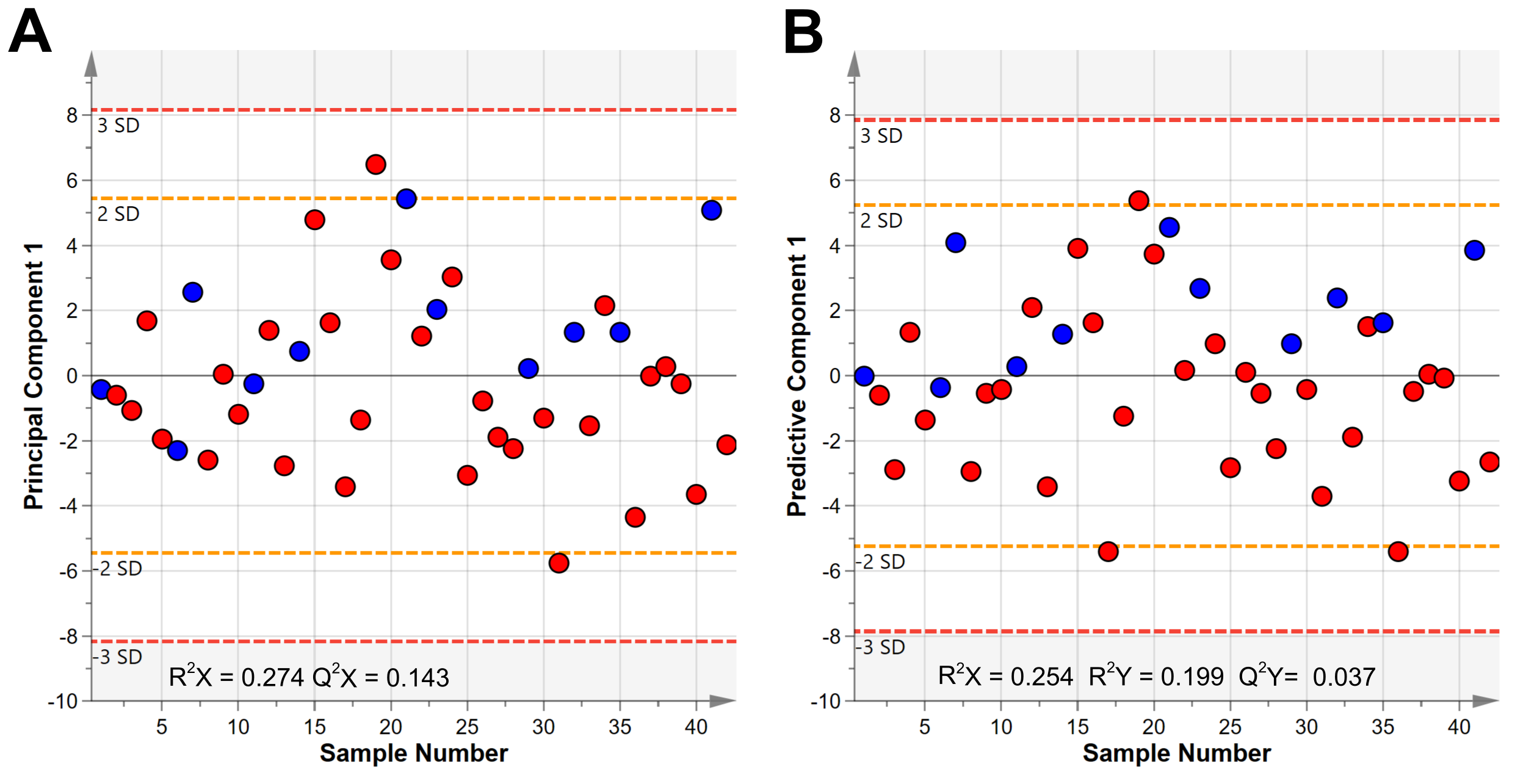
**

**Figure S1**. CSF Metabolite Integrals unable to distinguish between HAT Diagnostic Stages

Scores plot for PCA (A) and corresponding OPLS-DA (B), based on 27 metabolite integrals peaks, measured in CSF from HAT patients via ^1^H NMR spectroscopy. Each circle represents the metabolic profile from one patient, where early stage patients are shown in blue (n=11) and late stage patients in red (n=31). No clear separation between the two groups is apparent in either analyses, also evident from the low model predictive parameter values (Q^2^). Abbreviations: R^2^X, model fit parameter for variation in spectral data; R^2^Y, model fit parameter for variation in classifier data (for OPLS-DA); Q^2^X; model predictive parameter for spectral data in PCA; Q^2^Y, model predictive parameter for classifier data in OPLS-DA; SD, standard deviation.
