# Supplementary material for: Metabolic Profiling of Central Nervous System Disease in Trypanosoma brucei rhodesiense Infection
Source: J Infect Dis. 2017 Sep 12;216(10):1273–80. doi: 10.1093/infdis/jix466 (PMC5853393; doi:10.1093/infdis/jix466)
Supplement: Supplementary Figure_S2 [file jix466_suppl_figure_s2.docx]

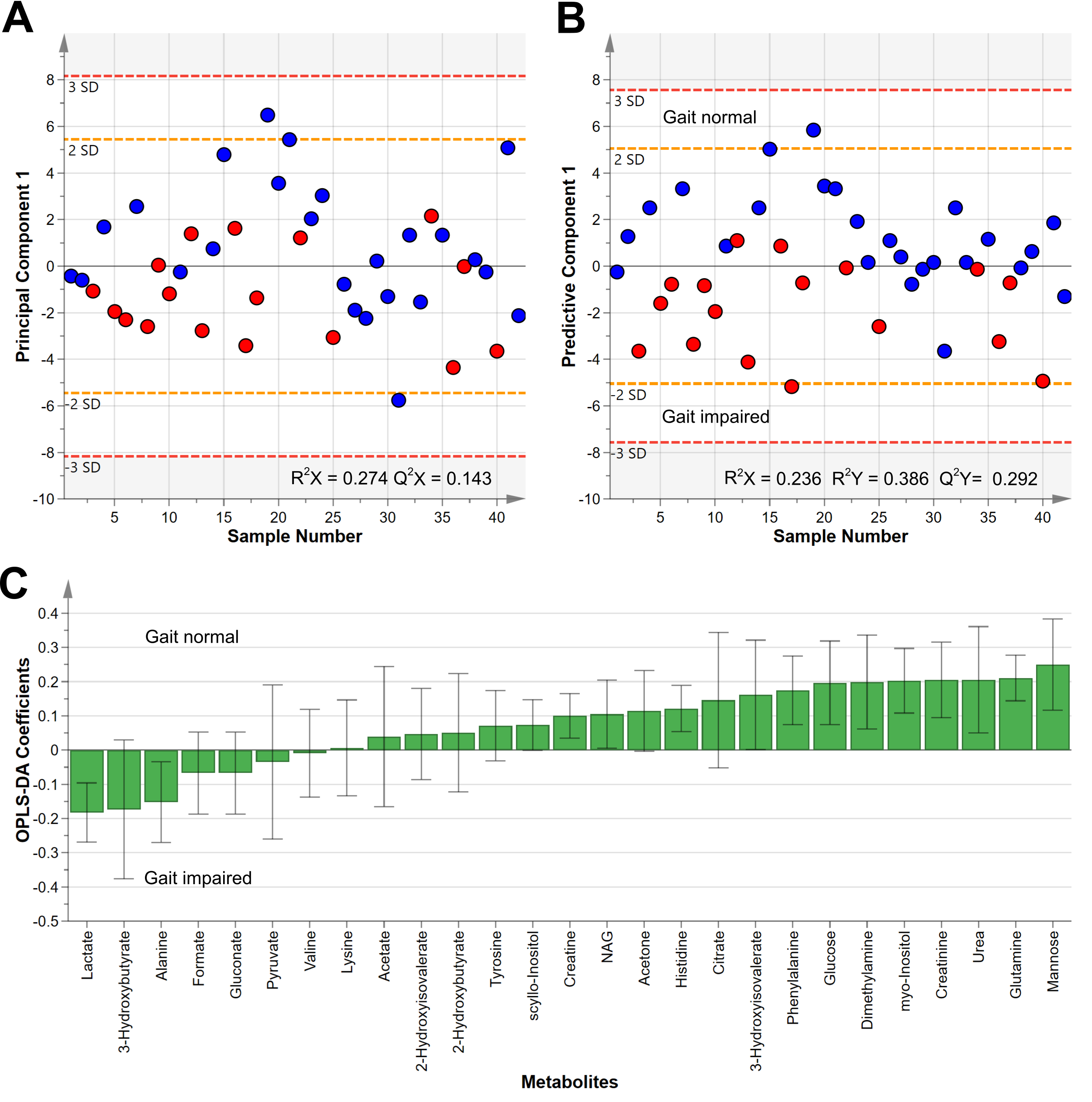


**Figure S2**. Metabolic Changes in CSF Associated with Impaired Gait

(A) PCA scores plot, comparing HAT patients with impaired gait (in red, *n*=17) vs. those with normal gait (in blue, *n*=25). (B) Cross-validated OPLS-DA scores plot and corresponding loadings plot (C), showing differences between the two groups. Abbreviations as described previously.
