# Supplementary material for: Metabolic Profiling of Central Nervous System Disease in Trypanosoma brucei rhodesiense Infection
Source: J Infect Dis. 2017 Sep 12;216(10):1273–80. doi: 10.1093/infdis/jix466 (PMC5853393; doi:10.1093/infdis/jix466)
Supplement: Supplementary Table [file jix466_suppl_s1_table.docx]

**S1 Table.** No significant differences in NMR metabolite levels were found between diagnostic stages in HAT

| **Metabolites** | **% Difference** | **P-value** | **FDR Corrected** |
| --- | --- | --- | --- |
| 2-Hydroxybutyrate | -7.1% | 0.2830 | NS |
| 2-Hydroxyisovalerate | -8.2% | 0.2580 | NS |
| 3-Hydroxybutyrate | 16.3% | **0.0053** | NS |
| 3-Hydroxyisovalerate | -21.3% | 0.0570 | NS |
| Acetate | 28.5% | 0.3990 | NS |
| Acetone | -13.8% | 0.0890 | NS |
| Alanine | 11.6% | 0.1480 | NS |
| Citrate | 1.6% | 0.9390 | NS |
| Creatine | -6.2% | 0.2130 | NS |
| Creatinine | -12.6% | 0.1120 | NS |
| Dimethylamine | -17.7% | 0.1780 | NS |
| Formate | 2.5% | 0.9200 | NS |
| Gluconate | 2.5% | 0.9200 | NS |
| Glucose | -26.3% | 0.1000 | NS |
| Glutamine | -17.7% | 0.0600 | NS |
| Histidine | -3.9% | 0.3600 | NS |
| Lactate | 30.6% | **0.0380** | NS |
| Lysine | -2.2% | 0.6370 | NS |
| Mannose | -31.3% | **0.0060** | NS |
| *scyllo*-Inositol | -19.5% | 0.4480 | NS |
| *myo*-Inositol | -5.6% | 0.5760 | NS |
| NAG | -10.7% | 0.1830 | NS |
| Phenylalanine | -8.1% | 0.1000 | NS |
| Pyruvate | -18.0% | 0.2580 | NS |
| Tyrosine | 0.8% | 0.5580 | NS |
| Urea | -20.3% | **0.0060** | NS |
| Valine | -3.4% | 0.8520 | NS |

Abbreviations: FDR, false discovery rate; *N*-acetyl glycoprotein; NS, not significant
